# Taxonomic and floristic novelties for *Echeveria* (Crassulaceae) in Central Michoacan, Mexico

**DOI:** 10.3897/phytokeys.75.9198

**Published:** 2016-11-25

**Authors:** Ignacio García-Ruiz, Dagoberto Valentín-Martínez, Pablo Carrillo-Reyes, Mihai Costea

**Affiliations:** 1 Instituto Politécnico Nacional, Centro Interdisciplinario de Investigación para el Desarrollo Integral Regional, Unidad Michoacán, Justo Sierra 28, Apdo. postal 109, C.P. 59510, Jiquilpan, Michoacán, México; 2 Escuela de Biología de la Universidad Michoacana de San Nicolás de Hidalgo, Morelia, Michoacán, México; 3 Universidad de Guadalajara, Departamento de Botánica y Zoología, Centro Universitario de Ciencias Biológicas y Agropecuarias, km. 15.5 carretera a Nogales, Predio Las Agujas, Zapopan, Jalisco 45110, México; 4 Wilfrid Laurier University, Waterloo, Canada, 75 University Avenue West, Waterloo, Ontario, N2L3C5, Canada

**Keywords:** Conservation, Echeveria, malpaís, morphology, ser. Gibbiflorae, ser. Valvatae, taxonomy

## Abstract

A new species, *Echeveria coruana*, is described and illustrated from the malpaís near San Andrés Corú, Michoacan, Mexico. The species belongs to series *Gibbiflorae* and the new taxon was compared with *Echeveria purhepecha* and *Echeveria patriotica*, with whom it shares the closest morphological affinities. Additionally, *Echeveria yalmanantlaensis* an endangered species from Sierra of Manantlán Biosphere Reserve, State of Colima, was also discovered near San Andrés Corú and is reported for the first time from the State of Michoacan. The conservation status of both species was (re)evaluated according to the criteria of the International Union for Conservation of Nature.

## Introduction

*Echeveria* DC. comprises *ca.* 140 species of which the majority (95%) have evolved in Mexico where the genus is characterized by a high degree of endemism (Uhl 1992, Thiede 1995, Meyrán and López-Chavez 2003, Pérez-Calix and Franco 2004, Vázquez et al. 2013). Among the infrageneric groups of this genus, ser. Gibbiflorae (Baker) Berger (sensu Walther 1972) is the third most diverse, being surpassed only by ser. Racemosae and ser. Nudae (Pilbeam 2008). It is noteworthy mentioning that the majority of new *Echeveria* species discovered in the last decade belong to ser. Gibbiflorae (García and Pérez-Calix 2007, Jimeno-Sevilla and Carrillo-Reyes 2010, Reyes and González 2010, Reyes et al. 2011a, 2011b, García and Costea 2014, Nieves-Hernández et al. 2014, Jimeno-Sevilla et al. 2015), which suggests an incomplete knowledge of the species diversity in this group.

The village of San Andrés Corú is located at *ca.* 12 km NE of the National Park Barranca del Cupatitzio, on the eastern side of the city of Uruapan, in the State of Michoacan (19°27.982'N, 101°56.644'W). This area is a part of the Trans-Mexican Volcanic Belt and has a particularly rich flora and vegetation consisting of a mixture of pine-oak and tropical deciduous forest elements (Rzedowski 1978). During the last years, systematic botanical explorations have been conducted to produce a floristic inventory of the malpaís surrounding San Andrés Corú. The malpaís (“badlands”) is a landform that consists of relict yet recognizable lava fields that exhibit various degrees of erosion and vegetation succession stages depending on their age (Neuendorf et al. 2005). In some of the field trips undertaken NW and W–SW of the village, an unknown *Echeveria* belonging to ser. Gibbiflorae was discovered. Also, growing in the same type of ecosystem, at *ca.* 6 km SE of San Andrés Corú, *Echeveria yalmanantlanensis* A. Vázquez & Cházaro, an endangered species of ser. Valvatae Moran previously known only from one population in the State of Colima (Vázquez et al. 2013), was also discovered. Thus, the first objective of this article is to describe the new species, which we named *Echeveria coruana*, and to explore its morphological affinities with other species of ser. Gibbiflorae. The second aim is to report *Echeveria yalmanantlanensis* as a new species for Michoacan.

## Materials and methods

In addition to herbarium specimens, flowers and leaves of *Echeveria coruana* and *Echeveria yalmanantlanensis* were fixed in FAA (Ruzin 1999) for morphological studies. Several living plants of both species were collected with soil and cultivated in Jiquilpan, Michoacán for further study. We examined the basic morphology of both fresh and fixed flowers under a Nikon SMZ1500 stereomicroscope equipped with a PaxCam Arc digital camera and Pax-it 7.8 software (MIS Inc., Villa Park, Illinois). For scanning electron microscopy (SEM), we used hexamethydisilazane (HMDS) as an alternative for critical dry point (Wright et al. 2011). Fixed flowers were dehydrated using a series of ethanol steps (70%, 80%, 95% and 100%; each step 10 minutes), immersed for 10 minutes in 1:1 ethanol: HMDS, and passed through three changes, each of 30 minutes in 100% HMDS. Samples were air dried and coated with 20 nm gold using an Emitech K 550 sputter coater. Micromorphological examination, measurements and pictures were taken at 10 kV using a Hitachi SU1510 variable pressure scanning electron microscope. Because only *Echeveria coruana* is described, micromorphological data for pollen and seeds are presented only for this species. Additional images than those provided in the article have been uploaded in Phytoimages (Nickrent et al. 2006 onwards).

## Results

The new species, *Echeveria coruana* belongs to ser. Gibbiflorae, which as summarized by Kimnach (2003), includes glabrous plants, acaulescent or with monopodial stems; leaves are medium-sized to large, commonly narrowed basally into a pseudo-petiole; inflorescences are paniculiform; bracts are similar to the leaves but smaller; pedicels may reach 10 mm in length; calyx has unequal sepals; corolla is large, up to *ca.* 13 mm long, pentagonal-conical in bud, cylindrical-urceolate to campanulate at anthesis, ± glaucous, often with carinate petals exhibiting a basal cavity on the inner side; nectaries are large, fleshy; styles whitish to dark-red or nearly black. *Echeveria coruana* possesses a distinctive characteristic encountered only in four other species of ser. Gibbiflorae — *Echeveria dactylifera* E. Walther (Walter 1972), *Echeveria novogaliciana* J. Reyes, Brachet & O. González (Reyes et al. 2011), *Echeveria marianae* I. García & Costea (García and Costea 2014), and *Echeveria rulfiana* Jimeno-Sevilla, Santana Mich. & P. Carrillo (Jimeno-Sevilla et al. 2015): the presence of corolla appendages at the base of antipetalous staminal filaments. However, it markedly differs from these species in having smaller leaf rosettes, a different leaf morphology, shorter inflorescences, cincini with fewer flowers, and shorter pedicels. Among all these species, *Echeveria coruana* has the shortest and most inconspicuous appendages. A detailed comparison of *Echeveria dactylifera*, *Echeveria novogaliciana* and *Echeveria marianae* was provided by García and Costea (2014), and *Echeveria coruana* can be easily contrasted with these species using the data included in Table 1. The most recently described species with corolla appendages, *Echeveria rulfiana*, differs from *Echeveria coruana* in its evidently caulescent habit and canaliculated leaves (Jimeno-Sevilla et al. 2015). Here we compared *Echeveria coruana* with two other species, *Echeveria patriotica* I. García & Pérez-Calix and *Echeveria purhepecha* I. García, which appear morphologically closer even if they do not possess corolla appendages (Table 1).

**Table 1.** Comparative morphology of *Echeveria coruana* with *Echeveria patriotica* (García and Pérez-Calix 2007) and *Echeveria purhepecha* (García 2011); “—“ indicates data not available for comparison.

Character	*Echeveria coruana* sp. nov.	*Echeveria patriotica*	*Echeveria purhepecha*
**Caudex**	Acaulescent or inconspicuous	Evident	Evident
Length × diameter (cm)	3–8 × 1–1.3	20 × 1–2.5	8–11× 1.2
**Rosette**			
Diameter (cm)	10–15	10–35	8–10
Position of leaves in rosette	Spreading	Spreading	Ascendant
**Leaves**			
Color	Light-green to dark green in the median part and apex	Green to reddish in the older leaves	Dark-green
Shape	Linear-oblanceolate to spathulate; apex acuminate, short mucronate	Oblong-obovate to spathulate; apex rounded, short mucronate	Oblong obovate; apex rounded, mucronate
Length (cm)	8–16	6–18	2–6.5
Width (cm)	2–2.5	3.5–11	1–2.5
**Inflorescence**			
Number of main axes	1–4	1–7	2–3
Length (cm)	28–65	20–100	15–30
Diameter at the base (cm)	0.3–0.5	1	0.3-0.6
Number of flowers/ cincinus	1–4(5)	1–8	1–7
**Bracts**			
Shape	Oblong-lanceolate	Oblong-lanceolate	Oblong-obovate
Length (cm)	0.8–7.2	1.1–12	0.8–3
Width (cm)	0.6–3	0.6–4	0.3–1.3
**Pedicel**			
Length (cm)	(1.3) 1.6–2.2	0.7–1	0.3–1.3
**Corolla shape at anthesis**	Cylindrical-urceolate	Cylindrical-urceolate	Cylindrical-urceolate to campanulate
Length (mm)	15–20	18–20	10–12
Width (mm)	7–9	10–14	ca. 8
**Sepals** (calyx)	Spreading to ± reflexed at anthesis	Spreading at anthesis	Addressed at anthesis
Shape	Triangular-lanceolate	Triangular-lanceolate	Oblong-elliptic to lanceolate
Length x width (mm)	6–9 × 3–4	9–14 × 3-6	4–9 × 2.5–3.5
**Petals**			
Shape	Lanceolate	Lanceolate	Oblong-lanceolate
Length × width (mm)	15–21 × 4–6.5	18–20 × 5-7.5	10–11 × 4
(External) color	Whitish-yellow at the base, light-yellow to orange in the median part and orange-reddish at the tips	White to cream white at the base, orange-reddish in the median part and scarlet-red at the tips	Scarlet-red or coral from the base to the tip
Appendages	(1)2 per antipetalous staminal filament; conical or dome-like, 0.2–0.3 mm long	Absent	Absent
**Nectaries**			
Length × width (mm)	1.8–2.2 × 0.8–1	3 × 1	1.2 × 0.5
Color	White-yellowish	Purple-red	Pale yellow
**Follicles**	5–6 mm long, erect to somewhat spreading	12–18 mm long, erect	ca. 5 mm long, erect
**Flowering**	Nov–Jan	Oct–Jan	Sep–Nov
**Geographical distribution**	Michoacan, Mpio. Ziracuaretiro: Malpaís de San Andrés Corú	Jalisco, Mpio. Mazamitla	Michoacan, Mpio. Nuevo Parangaricutiro
**Vegetation type**	Mixture of oak-pine and tropical deciduous forest	Oak and oak-pine forest	Oak-pine forest

### Echeveria coruana

Taxon classificationPlantaeSaxifragalesCrassulaceae

I.García, D.Valentín & Costea sp. nov.

urn:lsid:ipni.org:names:77158795-1

Figures 1 , 2

#### Diagnosis.

*Echeveria coruana* morphologically resembles most *Echeveria patriotica* and *Echeveria purhepecha*, with which it shares a similar flower morphology, but differs from both in having acaulescent or inconspicuous stems, acuminate leaves, and corolla appendages at the base of antipetalous stamen filaments. It differs from *Echeveria patriotica* in having smaller leaf rosettes, 10–15 cm in diameter, smaller inflorescences, 28–65 cm long, longer pedicels, (1.3) 1.6–2.2 mm and a narrower corolla, 7–9 mm in diameter. It can be distinguished from *Echeveria purhepecha* by the larger rosettes with spreading linear-oblanceolate to spathulate leaves, 8–16 cm long, and the larger corolla, 15–20 mm long, light-yellow to orange in the median part and orange-reddish at the tips of corolla lobes.

#### Type.

MÉXICO. Michoacán: Municipio de Ziracuaretiro, lado noroeste de San Andrés Corú; 19°28.116'N, 101°57.410'W; 1730 m; bosque de encino-pino alterado con huertas de aguacate; 27 Nov 2015; *I. García & M. García 9138* (holotype: CIMI!, isotypes: DAO!, ENCB!, IEB!, MEXU!, MICH!, WLU!).

#### Description.

*Perennial herb*, glabrous, acaulescent or with an inconspicuous caudex, 3–8 cm long and 1–1.3 cm in diameter; *rosette* lax, 10–15 cm in diameter with 15–18 leaves; *leaves* fleshy, light-green to dark green in the median part and apex, leaf blade linear oblanceolate to spathulate, 8–16 × 2–2.5 cm, 0.5–1 cm thick at the base, margin entire, occasionally with a thin, red-colored line, apex acuminate, short mucronate, base narrowed to form a pseudo-petiole, 2–3 cm long, corrugated ventrally; *inflorescence* paniculiform thyrse, 1–3 per rosette, 28–65 cm long and 0.3–0.5 cm wide at the base, with 1–4 secondary axes (cincinni), each with 1–4(5) flowers; bracts spiralled, adpressed, green to yellowish-red, oblanceolate to oblong-lanceolate, 0.8–7.2 × 0.6–3 cm, 1.2–1.5 mm thick in the median part and 7–8.5 mm at the base, base auriculate, soon caducous; pedicels (1.3–) 1.6–2.2 mm long and 2–3 mm thick; *calyx* gamosepalous, star-shaped, the tube 1–1.5 mm long, lobes green, unequal, 6–9 × 3–4 mm, spreading to somewhat reflexed at anthesis, triangular-lanceolate; *corolla* pentagonal-conical in bud, cylindrical-urceolate at anthesis, petals 15–21 × 4–6.5 mm, fused for 1–1.5 mm at the base, lanceolate, carenate, tips mucronate, erect to slightly deflexed, color whitish-yellow at the base, light-yellow to orange in the median part and orange-reddish at the tips; *nectaries* reniform, 1.8–2.2 × 0.8–1 mm, white-yellowish; *stamens* 10, 5 antipetalous, 9–16 mm long (including the anthers), with a pair of conical or dome-like appendages at the base, 0.2–0.3 mm long; episepalous stamens 5, 10–16 mm long (including anthers); *pollen* polymorphic, most abundant type is 3-colpate, oblate to oblate-spheroidal in equatorial view and triangular or round in polar view, 30–34 × 17–20 mm, less common grains are 3-colpate, prolate, 34–38 × 18–20 mm or 4-colpate, rectangular or spherical, 28–31 × 17–20 mm; in all pollen grains tectum is imperforate, scabrate; pollen grains eventually agglutinate into large masses; *ovary* with 5 apocarpous carpels, 9–11 × 0.3–0.4 mm; styles (including the stigmas) 4–5 mm long, red-purplish; *follicles* 5–6 mm long, erect to somewhat spreading; *seeds* numerous, oblong to obovate, light to dark-brown, reticulate, 0.5–0.65 × 0.2–0.25 mm; reticulum size 15–30 mm.

**Figure 1.**
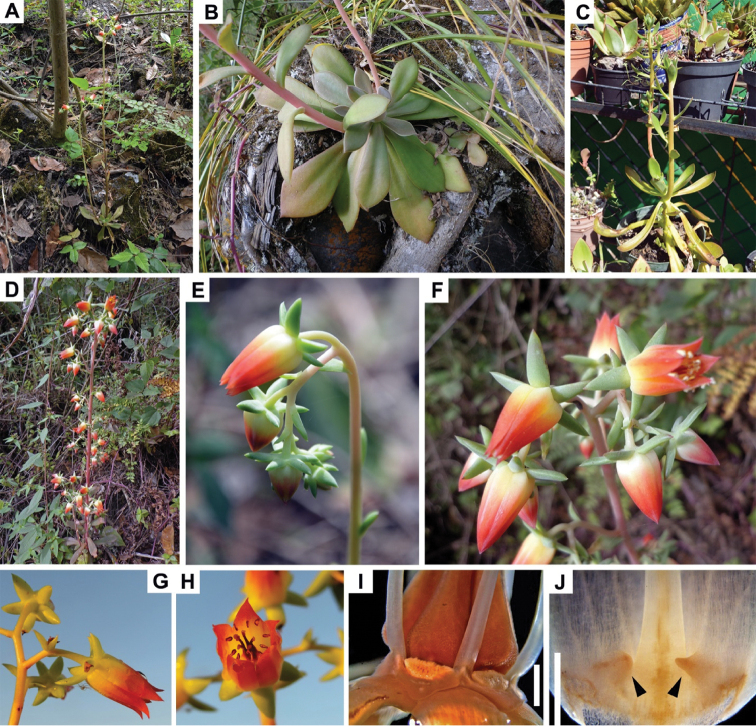
Habitat and general morphology of *Echeveria coruana*. **A** Habitat **B** Leaf rosette of type specimen **C** Developing plant (in cultivation) **D–F** Inflorescence **D** General view **E** Developing cincinus **F** Terminal cincinni **G–H** Flowers of type specimen viewed in the field from different angles **I–J** Flowers from type specimen fixed in FAA **I** Dissected flower (removed corolla) to show stamen bases and nectaries. **J** Conical appendages at the base of antipetalous staminal filaments (indicated with black arrows). Scale bars 1 mm.

**Figure 2.**
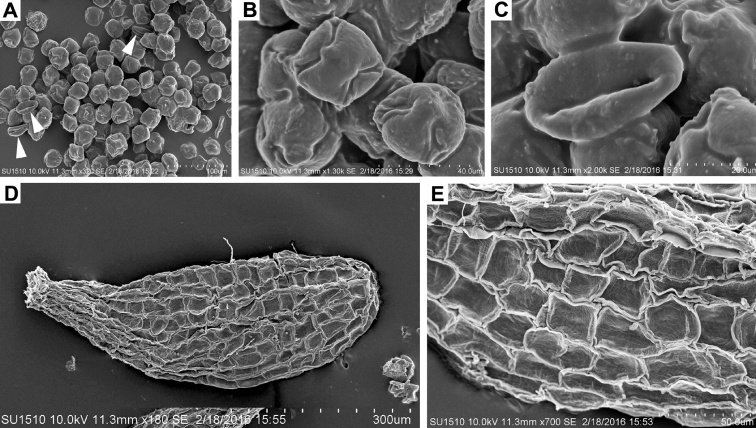
Scanning electron microscopy of *Echeveria coruana*, pollen and seed (*García & García 9138*). **A–C** Heteromorphic pollen grains; arrows indicate a few 3-colpate, prolate pollen grains **B** 4-colpate (left, upper) and 3-colpate pollen (right, down) grains **C** 3-colpate, prolate pollen grain **D–E** Seed morphology.

#### Discussion.

A detailed comparison of *Echeveria coruana* with *Echeveria patriotica* and *Echeveria purhepecha* is presented in Table 1. If these three species are related from an evolutionary point of view, the corolla appendages have evolved at least two times in ser. Gibbiflorae. The antipetalous stamen appendages of *Echeveria coruana* are considerably smaller and less complex than those of *Echeveria novogaliciana*, *Echeveria marianae*, *Echeveria dactylifera* and *Echeveria rulfiana*, the other species of ser. Gibbiflorae that are known to possess them (García and Costea 2014, Jimeno-Sevilla et al. 2015). García and Costea (2014) indicated that these appendages do not have a secretory function and their role may be to protect the nectar accumulated at the base of petals from pollinators lacking a specialized feeding apparatus. In *Echeveria coruana*, the appendages are too small to cover the corolla cavities in which nectar accumulates. Alternatively, if *Echeveria coruana* is evolutionarily related to these latter four species, the reduction of antipetalous corolla appendages in *Echeveria coruana* likely indicates the loss of this hypothetical nectar defense function. Corolla appendages at the base of stamens have also evolved in *Pachyphytum* (Walther 1972, Thiede and Eggli 2007), a genus that forms a sister clade to the remaining “Echeveria group” (Carrillo-Reyes et al. 2009). A molecular study for ser. Gibbiflorae with more extensive sampling that of Carrillo and et al. (2009) is necessary to understand the evolutionary relationships among the numerous members of this group (Walther 1972), including the several recently described species.

#### Ecology.

The new species grows in the understory of mixed pine-oak and tropical deciduous forest on volcanic basaltic rocky outcrops or small ledges. However, it has also been observed growing epiphytically on *Quercus* sp. The tree layer is dominated by *Quercus magnoliifolia* Née, *Ficus membranacea* C. Wright, *Juglans major* (Torr.) Heller, *Photinia microcarpa* Standl., *Bursera ariensis* (H.B.K.) Mc. Vaugh & Rzed., and *Clusia salvinii* Donn.; the most common shrubs are *Bursera bipinnata* (Sessé & Moc. ex DC.) Engl., *Montanoa bipinnatifida* (Kunth) C. Koch, *Montanoa frutescens* (Mairet) ex DC. and *Rhus terebinthifolia* Schltdl. & Cham. The herbaceous understory vegetation includes among others: *Arenaria lanuginosa* (Michx.) Rohrb, *Bonplandia geminiflora* Cav., *Tripogandra amplexicaulis* (Klotzsch ex C.B. Clarke) Woodson, Phaseolus acutifolius var. latifolius G.F. Freeman, and *Dryopteris maxonii* Underw. & C. Chr.

#### Phenology.

November to January.

#### Etymology.

The specific epithet derives from San Andrés Corú, the nearest village to the malpaís where the species was discovered. “Corú” in the local Purhépecha language means “a place where the quails sing”.

#### Conservation status.

*Echeveria coruana* is currently known only from three populations located at *ca.* 1–2 km from one another in the malpaís of San Andrés Corú. Although it is relatively common in the studied sites, it is threatened because of the increasing demand and exploitation of volcanic rocks in the area. Furthermore, the recent establishment of avocado orchards at elevations of 1670–1750 m has led to significant habitat loss in the area, and this practice is likely to continue in the future. Although it was not possible to use GeoCAT (Bachman et al. 2011) to calculate the extent of occurrence because of the reduced number of localities from which the species is known, we determined the area of occupancy, which was 8 square km (based on 2 km cells). Therefore, using the IUCN (2012) criteria B2 biii, we preliminarily categorize this species as Critically Endangered (CR). More research in the field will be carried out in the future to determine the best strategy to mitigate the above mentioned threats.

#### Additional specimens examined.

México. Michoacán: Municipio de Ziracuaretiro, Malpaís de San Andrés Corú, bosque de encino-pino, 1676 m, 1 Dec 2012, *D. Valentín 502* (CIMI!, EBUM!); Malpaís de San Andrés Corú, lado oeste-suroeste de San Andrés Corú, 1660 m, 29 Apr 2015, *I. García*, *D. Valentín* and *A. Fuentes 9078* (CIMI!).

### *Echeveria yalmanantlanensis* new for the flora of Michoacán

**Figure 3.**
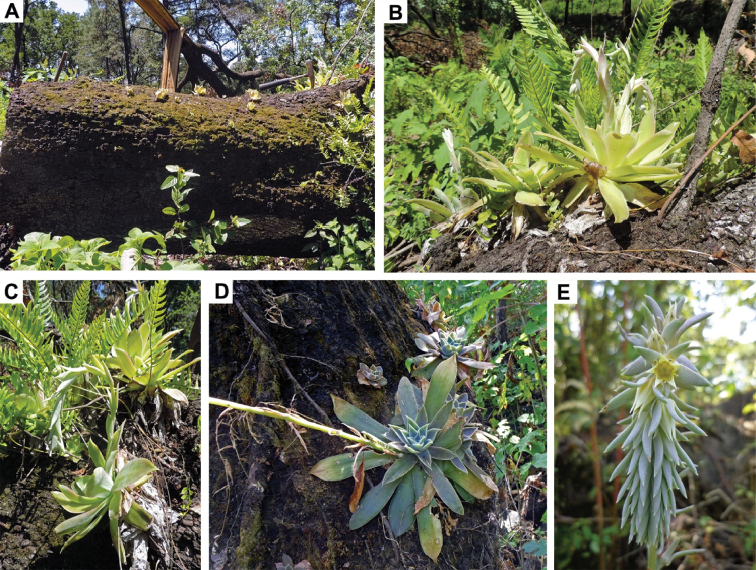
*Echeveria yalmanantlensis* in the new habitat from Michoacan. **A** General habitat view of young epiphytic plants **B–D** Leaf rosettes and developing plants **E** Inflorescence.

The exploration of the malpaís located at *ca.* 6 km SE of San Andrés Corú also led to an important floristic discovery: a new record of *Echeveria yalmanantlanensis* (Fig. 3). This species has been considered in danger of extinction and endemic to the Cerro Grande Massif, which is situated in eastern Sierra of Manantlán Biosphere Reserve, Municipality of Comala, State of Colima, where it is known from one single population (Vázquez et al. 2013). More than a decade of concerted explorations conducted by multiple botanists to find additional populations at the type locality, the adjacent volcanic areas, Sierra de Manantlán Central, and Nevado de Colima, have been unsuccessful (reviewed by Vázquez et al. 2013). Under these circumstances, it was totally unexpected to discover it in Michoacan at about 210 km from the type locality. This finding suggests a disjunct distribution of *Echeveria yalmanantlanensis*, which although rare may also be present at other localities in Central Michoacan.

**Habitat and phenology of *Echeveria yalmanantlanensis*.** In Michoacan, *Echeveria yalmanantlanensis* grows on volcanic rocks and occasionally as epiphyte in shady habitats that maintain sufficient humidity even during the dry season (Fig. 3). The vegetation at the new locality is very similar to that of *Echeveria coruana* (see above), consisting of a mixture of pine-oak and tropical deciduous species. The vegetation at the type locality in Sierra of Manantlán includes some elements of tropical deciduous forest at 1500 m above the sea level; however, the companion species indicated by Vázquez-García et al. (2013)—*Jatropha bartlettii* Wilbur, *Bursera macvaughiana* Cuevas & Rzed., and *Agave attenuata* Salm-Dyck. — have not been observed at the new site in Michoacan. Furthermore, at the original site, oak-pine forest vegetation elements were absent. The substrate at the original site in Colima State is calcareous, while in Michoacan it is volcanic. Also the epiphytic habitat observed in Michoacan (Fig. 3) was not reported from the type locality. The phenology is also somewhat different between the two sites: it extends to December at the new locality in Michoacan, while in Sierra of Manantlán plants were noted to flower from the end of July to the beginning of October. Only the climate is more or less similar at both sites, as it belongs to the type (A)C(w)(i´)(w_2_) (García 1988), semi-warm, sub-humid, with an annual average temperature between 18° and 22°C and characteristics intermediate between warm and temperate climates.

**Conservation status of *Echeveria yalmanantlanensis*.** Vázquez-García et al. (2013) proposed the inclusion of *Echeveria yalmanantlanensis* in the Mexican Endangered Species Act as an endangered species (Norma Oficial Mexicana, NOM-ECOL-059-2010). In Michoacan, only three populations with very few mature individuals (4–10) were found. An evaluation of the conservation status based on the geographical distribution using GeoCAT (Bachman et al. 2011) revealed that the extent of occurrence and area of occupancy (based on 2 km cells) in Michoacan are 62.01 km^2^ and 8 km^2^, respectively. Including the population from Colima and using the IUCN (2012) criteria, we provisionally propose an endangered status (EN) for this species. Like in the case of *Echeveria coruana*, the habitat of *Echeveria yalmanantlanensis* is threatened by the development of avocado plantations and exploitation of volcanic rock.

**Specimens examined.** MEXICO. Colima: Municipio de Comala (originally cited as Jalisco-Colima because of the proximity to the border between the two states), camino a Campo Cuatro, Cerro Grande, on a rock of a limestone slope, 1550 m, 26 Sep 2011, *J. Antonio Vázquez-García, M. Cházaro B. & J. Padilla-Lepe 9175* (holotype: IBUG!; isotype: NY!). Municipios Comala border, camino de Campo Cuatro a La Añilera, Cerro Grande, tropical dry forest, on a rock of a limestone slope, 1500 m, 18 Jul 2004, *Vázquez-García & Contreras 7830a, 7830b* (IBUG!). Michoacán: Municipio de Ziracuaretiro, Malpaís de San Andrés Corú, Bosque de Encino, epífita sobre *Quercus* sp., 1513 m, 22 Dec 2012, *D. Valentín 537* (CIMI, EBUM!); Malpaís de San Andrés Corú, bosque mixto con encinos, sobre rocosidades, 1510 m, 29 Apr 2015, *I. García & D. Valentín 9077* (CIMI!); 15 Sep 2015, *I. García & D. Valentín 9077* (CIMI!, WLU!).

## Supplementary Material

XML Treatment for Echeveria coruana
